# Protein Oxidation in Muscle Foods: A Comprehensive Review

**DOI:** 10.3390/antiox11010060

**Published:** 2021-12-28

**Authors:** Rubén Domínguez, Mirian Pateiro, Paulo E. S. Munekata, Wangang Zhang, Paula Garcia-Oliveira, Maria Carpena, Miguel A. Prieto, Benjamin Bohrer, José M. Lorenzo

**Affiliations:** 1 Centro Tecnológico de la Carne de Galicia, Rúa Galicia Nº 4, Parque Tecnológico de Galicia, 32900 San Cibrao das Vinas, Spain; rubendominguez@ceteca.net (R.D.); mirianpateiro@ceteca.net (M.P.); paulosichetti@ceteca.net (P.E.S.M.); 2 Key Laboratory of Meat Processing and Quality Control, Ministry of Education China, Jiangsu Collaborative Innovation Center of Meat Production and Processing, Quality and Safety Control, College of Food Science and Technology, Nanjing Agricultural University, Nanjing 210095, China; 3 Nutrition and Bromatology Group, Analytical and Food Chemistry Department, Faculty of Food Science and Technology, University of Vigo, 32004 Ourense, Spain; paula.garcia.oliveira@uvigo.es (P.G.-O.); mcarpena@uvigo.es (M.C.); mprieto@uvigo.es (M.A.P.); 4 Centro de Investigação de Montanha (CIMO), Instituto Politécnico de Bragança, Campus de Santa Apolonia, 5300-253 Braganca, Portugal; 5 Department of Animal Sciences, The Ohio State University, Columbus, OH 43210, USA; bohrer.13@osu.edu; 6 Facultade de Ciencias, Área de Tecnoloxía dos Alimentos, Universidade de Vigo, 32004 Ourense, Spain

**Keywords:** food quality, oxidative stress, meat and meat products, fish and fish products, protein cross-linking, carbonyls, analytical methods

## Abstract

Muscle foods and their products are a fundamental part of the human diet. The high protein content found in muscle foods, as well as the high content of essential amino acids, provides an appropriate composition to complete the nutritional requirements of humans. However, due to their special composition, they are susceptible to oxidative degradation. In this sense, proteins are highly susceptible to oxidative reactions. However, in contrast to lipid oxidation, which has been studied in depth for decades, protein oxidation of muscle foods has been investigated much less. Moreover, these reactions have an important influence on the quality of muscle foods, from physico-chemical, techno-functional, and nutritional perspectives. In this regard, the loss of essential nutrients, the impairment of texture, water-holding capacity, color and flavor, and the formation of toxic substances are some of the direct consequences of protein oxidation. The loss of quality for muscle foods results in consumer rejection and substantial levels of economic losses, and thus the control of oxidative processes is of vital importance for the food industry. Nonetheless, the complexity of the reactions involved in protein oxidation and the many different factors that influence these reactions make the mechanisms of protein oxidation difficult to fully understand. Therefore, the present manuscript reviews the fundamental mechanisms of protein oxidation, the most important oxidative reactions, the main factors that influence protein oxidation, and the currently available analytical methods to quantify compounds derived from protein oxidation reactions. Finally, the main effects of protein oxidation on the quality of muscle foods, both from physico-chemical and nutritional points of view, are also discussed.

## 1. Introduction

The oxidation of food proteins leads to reduced nutritional value, impaired food functionality, and in many cases loss of product quality [1,2,3,4,5,6,7]. Specific to muscle foods, which generally contain 17 to 25 percent protein in their raw/unprepared form [8], protein oxidation can be a hidden and significant form of deteriorative reactions. Protein oxidation is generally thought to be related to other oxidative reactions that occur in foods such as lipid oxidation and enzymatic reactions in which oxygen serves as a catalyst [9]. However, less attention has been provided to protein oxidation, as the pathways of oxidation are more complex, the variety of the oxidative products are greater, and the resulting effects (and discernible consequences) are often less noticeable by consumers.

Oxidative changes in the protein component of muscle foods are thought to occur when protein radicals are formed which can result in protein crosslinking, amino acid side chain modification, and/or protein fragmentation [2]. Certain amino acids have been discussed as being more susceptible to the formation of free radicals than others, certain proteins have been discussed as being more sensitive to protein oxidation than others, and some muscles have been shown to be more prone to noticeable differences in oxidation of certain proteins, such as myoglobin (for instance: color stable muscles versus color labile muscles) [2,3,4,7]. Nonetheless, protein oxidation is a complex process that warrants greater scientific research at the basic level.

Factors influencing susceptibility to protein oxidation for muscle foods can be described by both intrinsic factors and extrinsic factors. Intrinsic factors include animal species, origin of the animal (e.g., genetics and environmental factors during production), muscle type, and composition of the product and extrinsic factors include processing conditions, packaging conditions, and preparation techniques.

Laboratory techniques used to quantify the extent of protein oxidation in meat products include indirect analytical methods centered on measuring and evaluating the formation (and level of reactivity) of carbonyls, the formation (and level of reactivity) of carbonyl derivatives, the modification of amino acid side chains (specifically the formation of sulfhydryl groups), the formation of protein cross-links, and the aggregation or polymerization of proteins. There are certain limitations for each of these methods, as they are very time dependent, require a detailed protocol, and in some cases require the development of a working protocol. Perhaps the most common methodology for protein oxidation in the current research is focused on the analysis of carbonyl derivatives, and specifically the derivatization of protein carbonyls with 2,4-dinitrophenylhydrazine (DNPH) to form a DNPH complex. This complex can be identified and quantified with high performance liquid chromatography (HPLC) and mass spectrometry. The advantages of this method and other methods that focus on the analysis of carbonyl derivatives are that carbonylation occurs in the majority of oxidized proteins, whereas other methods, such as the quantification of sulfhydryl groups, are only useful for proteins with a significant number of amino acids with sulfur groups (i.e., methionine or cysteine) [7]. Nonetheless, laboratory techniques to measure protein oxidation in muscle foods seems to be an emerging and important area of emphasis for the food industry.

Protein oxidation has been shown to affect quality of muscle foods in a number of ways [3]. Specifically, studies have shown that protein oxidation has detrimental effects on color stability and textural properties during the refrigerated storage of meat products [2,3,4,7]. Perhaps a greater concern is the reduced nutritional value of protein foods when high levels of protein oxidation occur. When amino acid side chains are modified by reactive oxygen species, the metabolism of those amino acids will also likely be altered. This remains to be an under researched area of concentration in food science and human nutrition. In addition, the oxidized proteins and the end products derived from these proteins has also an important role in human health, since induced damage to human proteins and promote several diseases [10]. This is due to their potential mutagenic, carcinogenic, and neurotoxic activity [11,12]. However, the present manuscript focused on the main effects of protein oxidation on muscle foods quality, and not on the implications of intake of oxidized proteins in human health.

**Figure 1:**
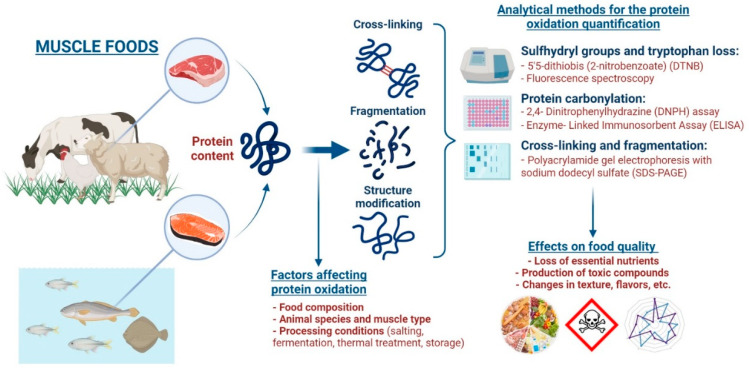
Schematic representation of the main mechanisms and factors that affect protein oxidation, different analytical methods for quantifying protein oxidation, and the detrimental effects of protein oxidation on muscle food quality.

Therefore, the objective is to address and provide a comprehensive review for the following areas of study related to protein oxidation—mechanisms of protein oxidation, factors affecting protein oxidation, analytical methods for the determination of protein oxidation, and effects of protein oxidation on food quality (Figure 1). This review will focus specifically on muscle foods; however, many of these general concepts can be applied to different sectors of the food industry.

## 2. Mechanisms of Protein Oxidation

Oxidation occurs ubiquitously in muscle foods, such as fresh and processed meat and fish products. Unlike lipid oxidation which is generally considered among the scientific community as well-described, protein oxidation is less recognized due to its imperceptible impact on flavor and appearance [13]. Protein oxidation, defined as a reaction causing the covalent modification of proteins, can cause irreversible damages to protein structure such as amino acid side chain modification, protein backbone cleavage, and protein cross-linkage, thereby resulting in undesirable changes of sensory quality, processing properties, and the nutritional characteristics of muscle foods [14]. Normally, the proteins found in foods can be oxidized by the direct attack of reactive oxygen and nitrogen species or the indirect induction caused by oxidative by-products from sugars and lipids [3,15]. Although studies have provided a general understanding of oxidation reactions in meat products, there is still a lack of systematic and in-depth literature focused on the basic principles of protein oxidation. Therefore, this review aimed to comprehensively discuss the mechanism of protein oxidation with an emphasis on the protein reaction process as well as the classification of protein oxidation (i.e., photo-oxidation, metal-catalyzed protein oxidation, and enzyme-catalyzed protein oxidation).

### 2.1. Protein Oxidation Process

**Figure 2:**
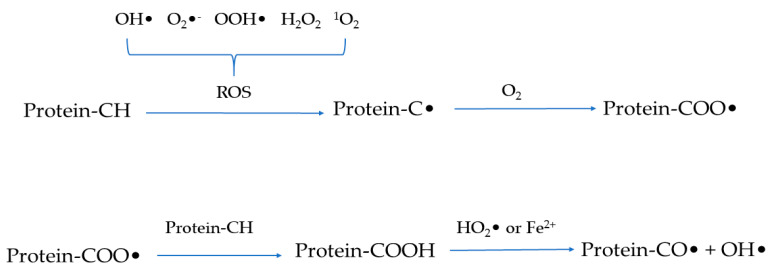
Schematic representation of the mechanism of protein oxidation.

Similar to lipid oxidation [9], the protein oxidation process also begins with the initiation stage of free radical formation and the generation of hydroperoxide before transitioning to the propagation stage of radical proliferation and transfer and then concluding with the termination stage which is summarized as the formation of non-reactive species [6,16]. In fact, oxygen exists in the form of triplet oxygen and cannot spontaneously react with protein molecules [6]. Therefore, the reactive intermediates such as reactive oxygen species (ROS) and reactive nitrogen species are required for protein oxidation to occur. ROS include free radicals such as hydroxyl radicals (^•^OH), superoxide anion radicals (O_2_^•^^−^), hydroperoxyl radicals (^•^OOH), and non-radical species such as hydrogen peroxide (H_2_O_2_) and singlet oxygen (^1^O_2_) [2,17]. Similarly, reactive nitrogen species are divided into free radicals and non-radical species as represented by peroxynitrite (ONOO^−^) and nitrite (NO_2_^−^), respectively. Among these reactive intermediates, free radicals are highly reactive and can directly react with protein molecules through hydrogen abstraction, coupling, oxygenation, and cleavage [18]. Briefly, as shown in Figure 2, ROS can trigger the removal of a hydrogen atom from protein molecules to produce a carbon-centered free radical (C^•^) which is then converted into alkylperoxyl radicals (COO^•^) under the action of oxygen. The formed COO^•^ can react with Fe^2+^ or hydrophilic superoxide radicals [3]. Alternatively, COO^•^ can dehydrogenate another protein molecule to form alkyl peroxides and then react with peroxy radicals or Fe^2+^ to form alkoxy radicals and hydroxyl derivatives, thereby triggering the chain reaction of protein oxidation. However, C^•^-C^•^ interactions generate carbon-carbon cross-linked derivatives under anaerobic conditions [4]. In addition, free radicals attack protein molecules mainly at three sites, including the backbone of peptides, aliphatic amino acid side chain groups, and aromatic amino acid side chain groups [19]. Furthermore, oxidoreductase, light, or radiation, as well as metal-catalyzed one-electron reduction reactions are well-acknowledged factors that induce (and accelerate) the generation of radicals [17,20]. During the process of protein oxidation, the central carbon group of the peptide backbone can be converted into alkylperoxyl and alkoxy radicals through the single-electron reduction, hydrogen abstraction, and oxygen addition of free radicals. Subsequently, the reaction can result in the cleavage of the peptide backbone or the formation of cross-linked protein derivatives through the α-amidation or diamide pathway. The side chain groups of aliphatic amino acids are susceptible to direct oxidation to form carbonyl compounds. Meanwhile, the side chain groups of aromatic amino acids are attacked by free radicals to generate reaction products which can be converted into aromatic derivatives and aromatic free radicals through hydrogenation and coupling reactions [14,21].

In addition to free radical-induced protein oxidation, lipid peroxidation and non-enzymatic glycosylation can indirectly mediate the oxidation modification of proteins. Lipid peroxidation is inevitable during the processing and storage of muscle foods, leading to the formation of many intermediate products such as alkylperoxyl radicals, alkoxy radicals, reactive carbonyl compounds, hydroperoxide, and others. Alkylperoxyl and alkoxy radicals can react at the peptide backbone and amino acid side chain groups while reactive carbonyl compounds can interact with the side chain groups of protein to form covalent cross-links [22]. In the meantime, hydroperoxide can react with protein to form an amide adduct via the ε-amino pathway. The reactive aldehydes produced from the lipid peroxidation reaction are mainly α, β-unsaturated aldehydes [23], which possess a high ability to induce protein oxidative denaturation [3,24]. On the other hand, non-enzymatic glycosylation also gives rise to the oxidation of proteins [25]. Additionally, ROS are involved in mediating the acceleration of non-enzymatic glycosylation, and the products produced by the glycosylation reaction can react with proteins, further leading to the occurrence of protein oxidation.

### 2.2. Protein Photo-Oxidation

Direct exposure of muscle foods and their products to light sources in the retail environment can present attractive color display that consumers find appealing and often times expect. However, this form of retail display can promote the photo-oxidation of protein. The occurrence of protein photo-oxidation mainly involves two mechanisms. Protein can be directly photo-oxidized through ultraviolet radiation as a result of the absorption by chromogenic groups [26,27]. The main chromogenic amino acids in proteins are tryptophan, tyrosine, and cysteine. The direct photochemical effects of protein are mainly led by the amino acid side chains, and the contribution of a specific side chain relies on its abundance in the given muscle food [26]. Additionally, this mechanism can result in electron transport and hydrogen abstraction in proteins to form molecules of the excited state species or radicals due to photo-ionization [7]. This can, in turn, give rise to protein damage and the alteration of molecular characteristics [20,28]. On the other hand, the singlet oxygen route is considered as another mechanism for photo-oxidative reactions. The singlet oxygen route refers to the conversion of triplet oxygen to singlet oxygen under the activation of photosensitizers (e.g., metal-free porphyrins) [29]. The reactive intermediates that are derived from singlet oxygen interacting with various amino acid side chain groups, which can trigger the oxidation of proteins.

### 2.3. Metal-Catalyzed Protein Oxidation

Metal-catalyzed protein oxidation is the most explored among the three protein oxidation types, which is likely because of its extensive occurrence in protein foods. The amino acid side chains from lysine, histidine, arginine, threonine, and proline are particularly susceptible to metal ion-catalyzed oxidation [14,30]. The superoxide anion generated from various pro-oxidation reactions is easily changed to H_2_O_2_ with different methods, including spontaneous reaction. In addition, the generation of a chelated compound is attributed to the combination of metal ions in the reduced state with amino acid residues at the metal binding sites of proteins and enzymes. The chelated compounds that are generated react with H_2_O_2_ to form highly active hydroxyl radicals. Afterwards, the hydroxyl radicals are particularly prone to attack the amino acid located at (or near) metal binding sites and further cause the formation of carbonyl derivatives.

Using the iron-catalyzed lysine residue oxidation as an example, after the reduction of Fe^3+^ (Iron (III); ferric state) to Fe^2+^ (Iron (II); ferrous state), iron combines with proteins to form coordination complexes. The H_2_O_2_ generated from the reduction of oxygen can bind to Fe^2+^ complexes to produce hydroxyl radical ions as well as Fe^3+^-protein complexes. After that, hydroxyl radicals take a hydrogen atom from the carbonyl group and join this with an ε-amino group to produce a carbon-centered radical. Meanwhile, the unpaired electrons are obtained to combine with Fe^3+^, which leads to the regeneration of Fe^2+^. Additionally, the ε-amino group is converted into imino derivatives. In the end, proteolytically-driven degradation of imino derivatives as well as the release of NH_3_ and Fe^2+^ are followed by the generation of aldehyde derivatives [31]. However, the reactions described above can only occur under the existence of free transition metal ions, which are scarce in the reaction system. It is plausible that the catalytic action of heme-containing proteins on those steps can be regarded as an alternative pathway for metal-catalyzed protein oxidation [32,33].

### 2.4. Enzyme-Catalyzed Protein Oxidation

In addition to non-enzymatic oxidation, an enzyme-induced pathway is also involved in protein oxidation. Usually, enzyme-catalyzed protein oxidation processes contain two steps including the catalytic generation of reactive radicals and their subsequent action on proteins. Specifically, food-related oxidases include glucose oxidase, which catalyzes the production of H_2_O_2_ from glucose [34], laccase, which produces phenol free radicals [35], lipoxygenase, which catalyzes the oxidation of unsaturated lipids to form hydroperoxides [36], and lactoperoxidase, which is involved with the formation of peroxy radicals [37].

## 3. Factors Affecting Protein Oxidation

In most storage settings, muscle foods are continuously exposed to an oxidizing environment; thus, the generation of reactive oxygen species (ROS) is a natural consequence. The imbalance between oxidizing agents and antioxidant molecules is responsible for oxidation and the corresponding degradation mechanisms of these products [38,39]. Protein oxidation is considered a novel concept within most food industries. It has been overlooked for many years in favor of lipid oxidation which has attracted the majority of the attention given to oxidative food reactions [2,40]. Protein oxidation in muscle foods occurs due to the direct interaction of proteins with ROS or with secondary products of other oxidative processes such as lipid oxidation [41]. Protein oxidation can be influenced by several different intrinsic factors (e.g., food composition, origin, animal species, muscle type, etc.) or several different extrinsic factors (e.g., salting, ripening, fermentation, thermal treatment, storage conditions, etc.), where processing conditions must be highlighted.

### 3.1. Intrinsic Factors: Composition of Muscle Foods

Protein oxidation is strongly connected with lipid oxidation. Proteins can react with each other, causing oxidation due to nitrogen or sulfur centers of reactive residues but proteins can also get covalently bound to secondary lipid oxidized products, such as aldehydes or reducing sugars, causing the modification of proteins [38,42]. So, the whole composition of the food, including proteins and lipids but also other compounds, such as carotenoids, phenolic compounds, or metals, can affect protein oxidation [2,38].

Myofibrillar proteins (i.e., myosin and troponin T), oxidizing lipids, and metal catalysts are the main initiators of protein oxidation [3,38]. Myofibrillar proteins in muscle foods can act as pro-oxidants giving rise to the initiation and acceleration of protein and lipid oxidation [3]. The oxidation of myosin results in disulfide and non-disulfide cross-links in myosin [2,3]. During lipid oxidation, peroxyl radicals can remove hydrogen atoms from proteins leading to the generation of radicals [2]. The inclusion of metallic compounds (for instance, compounds containing sodium, magnesium, aluminum, potassium, calcium, iron, copper, or zinc) is directly related to the oxidative stability of meat, since they can effectively promote the oxidation process [9]. Once initiated, the reaction of proteins in the presence of oxygen will lead to modifications of the amino acid side chains, formation of covalent intermolecular cross-linked proteins and peptide backbone scission or fragmentation [2,38,40].

In respect to the modifications of the amino acid side chains, these include thiol oxidation, aromatic hydroxylation, and the formation of carbonyl groups [3,38]. Cysteine and methionine are the most easily oxidizable amino acids since they contain sulfur atoms, whose anion is a strong nucleophile rich in electrons allowing them to act as antioxidants [3,4]. Other amino acids such as tyrosine, phenylalanine, tryptophan, histidine, proline, arginine, and lysine have been highlighted as highly susceptible to ROS action [2]. Furthermore, the presence of certain enzymes, such as calpain, has been linked to the oxidation of the myofibrillar proteins of muscle foods in the presence of calcium and its further degradation. Calpain activity will depend on other factors, such as pH, Ca^2+^ concentration, and temperature, among other intrinsic and extrinsic factors [3,43]. Additionally, the role of antioxidant compounds present in muscle foods has been assessed. For example, vitamin E has been demonstrated to exert a reduction in the number of oxidized proteins when exposed to oxidative inducing conditions such as irradiation [44].

### 3.2. Extrinsic Factors

Meat and fish products are frequently subjected to different processing techniques to gain functionality, improve the organoleptic properties of the product, and/or extend its shelf-life with preservation purposes [4] (Table 1). Some of the most common procedures related to processing of meat and fish products and their impact on protein oxidation will be explained in the following section.

#### 3.2.1. Salting and Curing

The addition of salt (NaCl; i.e., common table salt) to different foods, including fish and meat, has been a common practice for centuries with the aim of preserving food products, avoiding microbial spoilage, and improving palatability [4,45,46]. On the contrary, new market trends are changing to reduce the amount of salt (in the form of NaCl) in food products due to the associated risks of high levels of sodium consumption with hypertension and cardiovascular diseases [45,47,48].

**Table 1:** Studies evaluating extrinsic factors on protein oxidation in different muscle foods.

Product	Treatment	Effect	Ref.
Fresh beef tenderloin	Salting (NaCl) + TPP or mixture	Carbonyl content increase and tryptophan fluorescence intensity loss. Promoted formation of CML and CEL. Lower cooking loss and higher moisture content.	[46]
Pork meat Chinese dry sausages	Salting (NaCl) (2–4%)	Higher doses facilitate the protein oxidation, lipid hydrolysis and oxidation (higher lipase activity, higher TBARS values, and higher LOX activity).	[47]
Spanish ham	Dry curing (9–24 months)	Promoting role on lipid oxidation (higher TBARS values), major peptidyl PTMs and release of FAAs.	[49]
Pork ham	Dry curing—NaCl replacement with KCl, CaCl_2_ and MgCl_2_	No significant differences in acid lipase activity or lipid oxidation.	[50]
Mutton ham	Dry curing (0–180 days)	Increased proteins’ surface hydrophobicity, carbonyl content increase, and thiol content decrease.	[51]
BF & SM muscles ham	Salting + cold smoking + drying + ripening	Higher proteolytic, protein oxidation and total FAAs content in BF than in SM.	[52]
Beef jerky	Fermentation	Carbonyl content and TBARS increase in normal fermentation but at lower levels when starter cultures where used.	[53]
Harbin dry sausages	Fermentation	Carbonyl compounds formation and sulfhydryl loss decreased using mixed cultured starters.	[54,55]
Minced beef	Cold treatment (4 °C)	Carbonyl compounds increase. Free and total thiols decrease.	[56]
Obscure pufferfish (*Takifugu obscurus*)	Freezing-thawing cycles + LE + OC	Increased of sulfhydryl and tryptophan loss. Cross-linkage formation.	[57]
Dry-cured pork loins	Dry curing + freezing 18 °C, 5 months/thawing 12 h, 4 °C	Increased cross linkage through Schiff bases formation.	[58]
Pork loins	Aging (1ºC, 19 days) + fast-freezing (−80 °C)	Carbonyl content and TBARS increased through time. Increased lipid oxidation.	[59]
Pork sausages	Heat treatment	SH groups decrease. Carbonyls and SeS groups increase.	[60]
Bigeye tuna (*Thunnus obesus*)	Salting + Freezing	Synergistic effect on lipid oxidation: TBARS increase. Increased protein cross-linking formation.	[61]
Chicken leg and breast meats	Freezing (−7, −12, −18 °C)	Higher carbonyl compounds increase at higher temperatures. Decreased sulfhydryl loss at lower temperatures.	[62]
Yak meat	Air-drying	Carbonyl compounds increase. Sulfide bond content increase and total sulfhydryl group decrease.	[42]
Rabbit meat	Refrigerated vs. superchilled storage	Superchilled conditions showed TBARS decrease, lower metmyoglobin percentage, carbonyl content, and sulfhydryl loss.	[63]
*Rhea americana* meat	Air- (5 days) and vacuum- storage (28 days)	No evolution of protein and lipid oxidation when vacuum storage was used.	[64]
Pork patties	Guarana seeds extract incorporation	Carbonyl compounds and TBARS decrease.	[65]
Burger beef patties	*Rosa canina* L. extract incorporation	Tryptophan oxidation decrease. Increase formation of Schiff bases.	[66]
Duck breast muscle	Dietary curcumin supplementation	Carbonyl compounds and TBARS decrease. Free amino groups on myofibrillar protein increase.	[67]
Frozen-thawed duck breast muscle	Dietary resveratrol supplementation	Carbonyl compounds decrease and decreased sulfhydryl loss.	[68]

Abbreviations: Nε-(carboxymethyl)lysine (CML); Nε-(carboxyethyl)lysine (CEL); tripolyphosphate (TPP); Lipoxygenase (LOX); pulsed electric field (PEF); Peptidyl post-translational modifications (PTMs); free amino acids (FAAs); monosodium glutamate (MG); Biceps femoris (BF); Semimembranosus (SM); Light exposure (LE); Oxygen concentration (OC).

**Figure 3:**
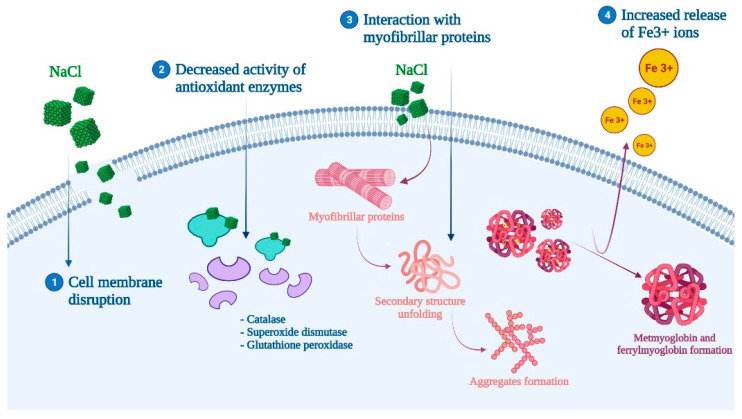
Proposed mechanism of action for salt (NaCl) and protein oxidation.

The inclusion of salt in muscle foods can also interfere with lipid and protein oxidation. However, there is controversy about its role, whereas while some studies point the oxidation promoting effect of salt, others have not found a significant relationship [47]. For those who support the pro-oxidant effect of salt, the responsible mechanism of action is thought to be related to (1) cell membrane disruption, (2) decreased activity of antioxidant enzymes, and (3) the increased release of Fe^3+^ ions and the formation of ferrylmyoglobin and metmyoglobin (Figure 3) [4,46,49]. Moreover, the inclusion of salt has been shown to promote protein and lipid oxidation when combined with other techniques such as drying or fermentation, or with the addition of nitrates and/or nitrites (which is defined as the curing process) [4,47,69]. For instance, curing has demonstrated the ability to affect protein oxidation and proteolysis, which can produce unpleasant effects on the organoleptic properties of further processed meat products [52] (Table 1). In this same perspective, the role of other techniques or pretreatments coupled with salt inclusion must be considered. A recent study assessed the effect of pulsed electric field (PEF) pretreatment on sea bass. PEF can cause a temperature increase and, therefore, an enhanced production of free radicals that lead to protein and lipid oxidation [45]. More specific studies have revealed that NaCl directly affected myofibrillar proteins by irreversibly unfolding their secondary structure, rupturing hydrogen bonds, and causing hydrophobic interactions to occur, while also promoting the formation of aggregates [70].

On the other hand, several non-sodium-containing alternatives have been proposed to replace NaCl, totally or partially, in food processing [71,72]. Specifically, other salts with similar chemical structure such as KCl, CaCl_2_, and MgCl_2_ have been evaluated as replacements for NaCl [73,74,75]. However, the functional and sensorial characteristics of these compounds in muscle food formulations are often not expected to be equivalent to NaCl, and their effects on protein oxidation need to be further studied [50,69].

#### 3.2.2. Fermentation

Fermentation is a processing technique that, when used for muscle foods, consists of treating meat or fish with specific microorganisms (e.g., bacteria or yeast) in an anaerobic setting and then drying or dehydrating the product. It is critical for fermented muscle food products to be processed under highly controlled conditions. The result of fermentation can increase flavor intensity and protect the product from spoilage for a long period of time, as well as provide several nutritional benefits, such as probiotic effects [4,76].

During the fermentation process, lipolytic and proteolytic enzymes from bacteria are responsible for the degradation of the meat or fish product, along with other changes such as the reduction of nitrates to nitrites, pH decrease, and oxidative phenomena [77]. In the case of muscle foods, fermentation can be spontaneously initiated by wild microorganisms or by starter cultures [4,53]. Several different studies have suggested the promoting effect of fermentation over protein oxidation while others have shown a delay of the process. A recent study showed that even though fermentation promoted protein oxidation, the effect was lowered when *Lactobacillus sakei*, *Pediococcus acidilactici*, and *Lactobacillus fermentum* were used, causing the inhibition of protein and lipid oxidation and changing the meat composition in terms of flavor and volatile compounds [53] (Table 1). Similar results were obtained when using a mixed starter culture of *P. pentosaceus*, *L. curvatus*, and *Staphylococcus xylosus*, which reduced the formation of carbonyl compounds and the loss of sulfhydryl groups, which resulted in improved flavor characteristics [54] (Table 1). The effect for protein oxidation is generally thought to result from the individual characteristics of the involved microorganisms: while some lactic acid bacteria can produce H_2_O_2_, a strong oxidizer, others can present neutralizer or antioxidant enzymes such as catalase or superoxide dismutase [54,55,78].

#### 3.2.3. Thermal Treatments

Extrinsic factors related to thermal treatment are referred to as procedures conducted at low temperatures (freezing or other forms of cold processing) or elevated temperatures (cooking or other forms of thermal processing). Initially, the crystallization caused by freezing can produce cell rupture and cryo-concentration of pro-oxidant compounds and oxidative enzymes that accelerate protein oxidation [4,79]. Holes and sponge-like texture in muscle foods can be produced by crystallization, increasing the surface exposed to oxygen [57] (Table 1). Moreover, the release of the heme or non-heme iron has been observed due to freezing, as a result of the oxidative cleavage of the porphyrin ring of myoglobin, leading to increased oxidation [79,80].

The effects of freezing–thawing cycles have been jointly assessed with light exposure and oxygen concentration. These factors promoted an increase of sulfhydryl and tryptophan loss that was more pronounced when the number of freezing cycles increased, so freezing was selected as the most critical factor [57]. Other studies have researched the effects of cooking and chilling after frozen storage. In this case, depending on the selected muscle, different values of protein oxidation products (α-aminoadipic and γ-glutamic semialdehydes) and heme iron content were obtained. Pre-freezing showed a remarkable impact on cooked hams [81,82]. Additionally, the presence of enzymes such as catalase and superoxide dismutase, polyunsaturated fatty acids, and lipid-protein interactions have shown influence on protein oxidation [62,81] (Table 1).

On the other hand, heat treatments and cooking have also been shown to affect protein oxidation and the quality of muscle foods. Elevated temperatures cause denaturation of proteins; for myoglobin, this process occurs at temperatures of approximately 55 °C, which results in the release of heme iron [4,83]. The connection between the oxidation of lipids and proteins has been assessed at different storage temperatures showing a wide range for oxidizing degrees and thresholds. Higher temperatures generally increased the carbonyl content of sarcoplasmic and myofibrillar proteins [84]. Similarly, a recent study evaluated protein oxidation in different luncheon meat products both before and after in vitro gastrointestinal digestion. The results showed that before and after digestion, protein carbonyl content was higher in raw-cooked and precooked-cooked products. Authors considered cooking and processing to be one of the main factors affecting protein oxidation in the selected products [85].

#### 3.2.4. Storage Conditions

The quality of muscle foods can be greatly affected by storage conditions, such as temperature and time, which can range from hours to months [84]. These two factors are strongly connected (for instance, longer times of storage are achieved when using lower temperatures) [63] (Table 1). Packaging conditions also influence resistance to protein oxidation. Anaerobic vacuum storage showed reduced levels of carbonyl formation and the absence of metmyoglobin oxidation compared to aerobic storage, preserving the meat properties for up to 28 days in refrigerated storage conditions [64] (Table 1). Other techniques such as the application of high pressure, magnetic fields, or electrostatic fields have been used for improving the quality and shelf-life of meats intended for prolonged periods of storage [4,80]. Low voltage electrostatic fields have been shown to be successful in reducing total carbonyl content and sulfhydryl content loss in prepared beef steaks when stored in frozen conditions for long periods of time [80].

Further approaches have been considered for counteracting protein oxidation. The inoculation of uncooked beef with *Lactobacillus plantarum* demonstrated a protective effect against the oxidative stability of proteins and lipids when beef was stored at 4 °C for 10 days [86]. The modification of the animal’s diet with the supplementation of bioactive compounds such as curcumin or resveratrol has also been shown to be a successful option to decrease protein oxidation in meat products [67,68] (Table 1). Lastly, most new trends are directed to the incorporation of natural extracts rich in phenolic compounds to inhibit or delay protein oxidation in muscle foods [65,66] (Table 1).

## 4. Analytical Methods for the Protein Oxidation Quantification

As explained in earlier sections, a significant level of complexity in reactions and interactions exist during protein oxidation, and there are a wide variety of factors that influence this process. Thus, it is almost impossible to develop a unique technique to evaluate protein oxidation in muscle foods. Considering this situation, numerous analytical techniques that target different markers of protein oxidation have been developed. Some of the most remarkable changes caused by this oxidative process include the loss of protein components, such as tryptophan and sulfhydryl groups, and the formation of oxidation products, such as the production of protein carbonyls and cross-links [2,4]. These indicators can be evaluated using different methodologies, which will be mentioned in this section. For food analysis, more than one measurement of protein oxidation is performed to guarantee detailed conclusions of the oxidative damage. In Table 2, different studies assessing diverse protein oxidation markers in muscle foods have been compiled.

### 4.1. Determination of Protein Carbonylation

**Figure 4:**
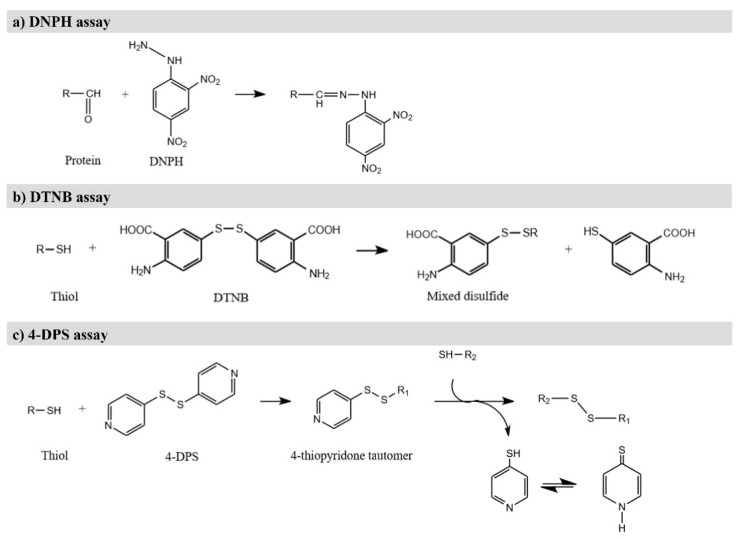
(**a**) Derivatization of carbonyl group with DNPH; (**b**) Derivatization of thiol group with DTNB; (**c**) Derivatization of thiol group with 4-DPS.

Protein carbonyls are formed in protein due to the oxidative deamination of alkaline amino acids, which include lysine, arginine, and proline [87]. Different methods have been employed to determine protein carbonylation, including the 2,4- Dinitrophenylhydrazine (DNPH) assay, which is one of the most convenient and common methods used in food analysis due to its simplicity and its low cost [3,4,101]. In this method, DNPH reacts with the carbonyl groups of proteins in acidic conditions to generate hydrazones (Figure 4a), which are quantified spectrophotometrically at 370 nm. Generally, the results are expressed as nanomol of carbonyl per milligram of protein, using the adsorption coefficient for the protein hydrazones [4,101]. This method has been successfully employed in numerous muscle foods, such as chicken breast [87], fermented sausages [89], dry cured ham, cooked sausage and other meat products [90], sturgeon fillets [97], house mackerel [93], and Pacific white shrimp [94] (Table 2). However, this technique has several drawbacks, such as lack of specificity and overestimation of carbonyls, both of which may lead to erroneous results [58,102]. In this sense, a shift in the absorbance wavelength from 370 to 450 nm has been reported to reduce the interferences with free DNPH [4].

To address the disadvantages of the DNPH assay, other methods to detect protein carbonyls have been developed, such as Enzyme-Linked Immunosorbent Assays (ELISA) and immunoblot methods. However, although these methods are highly sensitive, to our knowledge, they are not frequently employed in food analysis due to assay complexity, handling time, and cost per analysis in comparison with other techniques [102]. Several studies have developed alternatives to the DNPH assay that have been successfully tested in muscle foods. For example, Cropotova and Rustad [103] designed a fluorescence microscopy assay, using fluorescent labelling by coumarin-hydrazide. The method was tested in minced fish fillets. Authors observed that the results were comparable of those obtained using ELISA [103].

Protein carbonyls can be quantified by their total content by the DNPH assay and other techniques, but it is also possible to determine specific carbonyl compounds, such as α-aminoadipic (AAS) and γ-glutamic (GGS) semialdehydes. These compounds are produced by metal-catalyzed reactions that oxidize alkaline amino acids: lysine in the case of AAS, and arginine and proline residues in the case of GGS. Later, the aldehyde group of semialdehydes might be oxidized to an acid, leading to the formation of diacids. Aminoadipic acid (AAA) is generated from AAS, while glutamic acid is formed from GGS. The last one is not distinguishable from the natural glutamic, so it cannot be used as an oxidative marker [101]. Briefly, the quantification of AAS, AAA, and GSS is carried out using reductive amination by aminobenzoic acid and acid hydrolysis to release the products, and then they are analyzed by liquid chromatography coupled to fluorescence detection [95,100,102] or mass spectrometry [98,104]. This determination has been considered as a reliable protein oxidation marker for the analysis of different samples, including muscle foods such as fermented sausages [89], porcine patties [95], sturgeon fillets [98], and other diverse meat products [90], for instance (Table 2).

### 4.2. Detection of Loss of Sulfhydryl Groups and Loss of Tryptophan

The thiol group of cysteine is very sensitive to oxidation by ROS, leading to the formation of various oxidized products (for instance sulfenic acid or sulfinic acid) and the formation of disulfide cross-links [2]. Thus, the reduction of thiol content is an oxidative damage factor in muscle foods. The most common method to estimate this content is using Ellman reagent or 5′5-dithiobis (2-nitrobenzoate) (DTNB) as a derivatization agent to form a disulfide bond with free thiol groups (Figure 4b). This reaction releases a thiolate ion, which is colored and has a maximal absorbance of visible light at 412 nm [4]. This determination accounts for the total thiol content, so the free thiol content should be determined in unreduced proteins. The loss of thiol groups is then calculated by difference [102]. The DTNB assay has been widely applied in muscle foods, such as rabbit meat [96], sturgeon fillets [97], horse mackerel [93], and silver carp [99].

Loss of thiol groups can also be determined with 4,4′-dithiodipyridine (4-DPS) (Figure 4c), which has better solubility and smaller molecular volume than DTNB [101]. Using this reagent, protocols set the absorbance at 324 nm. Several studies have chosen this method to evaluate the thiol content of chicken breasts [87], different jerky chicken preparations [100], pork sausages [91], silver carp [99], and Pacific white shrimp [94] (Table 2).

Regarding tryptophan, the chemical structure of this compound has an aromatic ring, which is responsible for the natural fluorescence emitted at 350 nm when it is excited at 280 nm. Changes in the fluorescence of this amino acid are used to assess physio-chemical changes in proteins. In fact, the reduction of fluorescence has been linked to oxidative degradation of tryptophan and its transformation into radicals [58,95]. Loss of tryptophan is evaluated by fluorescence spectroscopy, a technique that offers several advantages, such as simplicity and rapidity, and is a solvent-free procedure [95]. In the literature, tryptophan loss has been evaluated in muscle foods such as jerky chicken [100], porcine and beef patties [66,95], ready-to-eat chicken patties [88], and rabbit meat [105] (Table 2).

### 4.3. Assessment of Cross-Linking and Fragmentation of Proteins

Free radicals produced during protein oxidation may cause the formation of cross-linked proteins, disulfide bonds, and Schiff base. In addition, these compounds may react with the peptide chains and cause fragmentation of the protein [102,106]. Specifically, cross linking, fragmentation, and the disruption of quaternary structure of proteins cause changes in the molecular weight patterns, which can be easily evaluated by separation of proteins by polyacrylamide gel electrophoresis with sodium dodecyl sulfate (SDS-PAGE) [4,107]. This technique is the most used to evaluate this protein oxidation manifestation and has been employed in rabbit meat [96], ground beef [99], sturgeon fillets [97], and rainbow trout [92], among others (Table 2). In some studies, immunoblotting was performed after SDS-PAGE to identify and better quantify the oxidized proteins [92,93].

As mentioned before, disulfide bonds are formed due to the oxidation of cysteine’s thiol group and they are estimated as half of the difference between total and free thiol content in non-reduced and reduced filtrates, respectively [100]. Some examples of studies that determined the disulfide bonds in muscle foods evaluated ground beef [99], jerky chicken [100], and ready-to-eat chicken patties [88] (Table 2).

Regarding Schiff bases, they derive from the reaction of amino groups from alkaline amino acids (e.g., lysine) and protein carbonyls (mainly AAS). Schiff bases emit fluorescence, so they are easily measured using fluorescence spectroscopy. The emission spectrum is recorded between 400 nm and 500 nm wavelength with and excitation at 350 nm, and the results obtained are expressed as fluorescence intensity units emitted by protein oxidation products at 450 nm [58,87]. In the literature, different studies have carried out this analysis to evaluate protein oxidation in muscle foods such as chicken breast [87], dry-cured loins [58], pork and beef patties [66,95], and sturgeon fillets [98] (Table 2).

## 5. Effects of Protein Oxidation on Food Quality

Several studies on protein oxidation have focused on the medical area because oxidized proteins and their products (such as heterocyclic aromatic amines or advanced-glycation end products) induce damage to human proteins, and consequently may promote certain diseases [10]. Some authors reported that the heterocyclic aromatic amines intake is associated with a variety of diseases, such as nonalcoholic fatty liver disease and neuronal damage, which promotes Parkinson’s, Alzheimer’s and other neurodegenerative diseases. This is due to these compounds having a neurotoxic, mutagenic, and carcinogenic activity [11,12]. Thus, international authorities recommended reducing human exposure to these compounds [108]. In addition, the accumulation of advanced glycation end products was also related to the promotion of several chronic diseases, such as diabetes, atherosclerosis, tumors, and Alzheimer’s disease [109,110]. Protein oxidation plays a vital role in the formation of these compounds, since protein oxidation products promoted the movement of precursors [10]. Thus, protein oxidation can generate active carbonyl compounds, which can be involved in the Maillard reactions and generate heterocyclic aromatic amines [108] and other advanced glycation end products [10]. In this sense, the free radicals derived from protein oxidation promote the heterocyclic aromatic amines since the reactions of protein oxidation and Maillard are interrelated and share common chemical mechanisms and intermediates [108]. In fact, a very recent study found a direct and positive correlation between oxidation, precursors, and end products [10]. However, the implications of protein oxidation on human health were discussed in several recent reviews [5,6]. Therefore, this topic is outside the scope of this review, which focuses on the consequences that these degradative reactions have on the quality of muscle foods.

As commented, in addition to the health implications, it is evident that the modification of proteins due to oxidative degradation produces significant effects on the physico-chemical properties and functionality of food proteins [111], which change the foods quality [112]. However, the importance of protein oxidation on the deterioration of muscle food quality has only been partially investigated [2]. Among all possible reactions, modifications of amino acid side chains such as thiol losses or the formation of carbonyls are the two most important changes that influence the quality of muscle foods [111]. In this sense, histidine was considered the main contributor of the increase in the formation of carbonyls [112]. Additionally, oxidative modifications also can lead to altered protein charges, which offer a new perspective to comprehend the mechanisms that protein oxidation generate on food quality [112]. Another important consequence of protein oxidation is the formation of protein cross-linking with covalent bonds within a protein or between proteins [2,111].

Therefore, it is expected that protein oxidation results in changes of the sensory quality, techno-functional properties, and nutritional characteristics of muscle foods [14,88]. All these modifications could be grouped into two main categories. One would be the influence of protein oxidation on the physico-chemical characteristics (and therefore the techno-functional properties) of foods, and another would be the main effects on the nutritional quality of muscle foods.

Among all physico-chemical properties of muscle foods, color, flavor, and texture are perhaps the most important attributes that determine, and affect, consumer acceptability [112]. Consequently, the microscopic changes that occur during protein oxidation could exert an enormous impact on the quality of muscle foods at the macroscopic level [111].

Generally speaking, it is accepted that oxidative reactions produce a negative effect on the texture of muscle foods by causing an increase in hardness and shear force values and a decrease in tenderness levels [88,111]. In this regard, the oxidative modification of proteins which implies the spatial arrangement, the formation of protein cross-linking, and the modification of protein net charges have a direct impact on the food texture [111,113]. Among all explanations, the increase of hardness and toughness of foods are normally related to two main effects: the increase of protein cross-link structures [111,114,115,116] and the reduction of proteolytic enzyme activity [6,101,111,117].

An increase in the toughening of muscle foods is often accompanied by the formation of protein cross-link structures; thus, it can be assumed that the myofibril cross-links are directly related to the textural changes in foods [111,114,115,116]. In contracted sarcomeres, an altered structure that contains greater overlap of thick filaments (i.e., myosin) and thin filaments (i.e., actin) will allow for the formation of more disulfide bonds, leading to a texturally harder product when compared with the relaxed muscle structure in living animals [112]. Among all cross-link reactions, the formation of disulfide through the oxidation of cysteine thiol groups, the formation of dityrosine through the interaction of two tyrosine radicals and the reaction between carbonyls (both, from lipid or protein oxidation products), and the generation of lysine ε-amino groups are the most common [2,112,117]. The relationship between food toughness and myofibrillar protein cross-linking has been shown by multiple authors in different muscle foods [115,116]. In fact, intra- and inter-molecular cross-linking is the main cause of structural changes in muscle foods, and therefore is thought to be the primary cause of loss of functionality in oxidized proteins [3,117].

However, available literature also suggests another possible explanation for the effect of protein oxidation on changes in food texture. Some researchers have indicated a relationship between the changes in muscle food texture with the oxidative degradation of proteins and therefore, the decrease in the activity of proteolytic enzymes [3]. It is well known that post-mortem proteolysis is a vital process in meat tenderization and is the primary mechanism involved with meat becoming tenderer following aging at refrigerated temperatures. Proteolytic enzymes are responsible for this tenderization, and among them, calpains are the main enzymes that are responsible. These enzymes require the transfer of electrons between cysteine and histidine residues. However, if these residues are oxidized, then the calpain activity is inhibited, and thus proteolysis (and muscle food tenderization) is reduced [112,115,118]. Nevertheless, it is important to consider the degree of oxidation when discussing the relationship between tenderness and proteolytic activity, since extensive oxidation leads to more compact protein structures, while moderate oxidation may unfold proteins and actually make them more accessible for enzymes to break down structural proteins. Therefore, the increase in toughness of muscle foods that have experienced high levels of protein oxidation is generally attributed to protein cross-linking rather than reduced proteolysis [112,115].

Regarding color, it is well known that the chemical and oxidative state of myoglobin are vital factors that determine the color of muscle foods (e.g., color stability of red meat) [9,111]. Myoglobin is a globular protein (consisting of 153 amino acids and a prosthetic heme group) found in muscle. This heme group gives myoglobin and its derivatives their distinctive color [119]. However, this protein is highly susceptible to oxidation, which produces a brown-colored oxidative state that often results in an association with food spoilage by consumers. The oxidation of ferrous-oxymyoglobin (Fe^+2^) to ferric-metmyoglobin (Fe^+3^) is responsible for the discoloration of meat, fish, and their products [120]. Additionally, it was stated that myoglobins in fish are more readily oxidized than mammalian counterparts [119]. The visual appearance change of this oxidative process in muscle foods is a change from a bright red color to a dull brown color, while the most obvious instrumental color change is a decrease in redness (a* parameter) and an increase in yellowness (b* parameter) [121]. In this sense, authors have shown a direct relationship between myoglobin autoxidation and meat color stability [122]; thus, a direct impact of protein oxidation on the color of muscle foods is often assumed. Similarly, other authors also correlated the myoglobin oxidation state (and also protein oxidation measured as total content of carbonyls) with sensory discoloration [120] and instrumental color changes in ground meat products [65,120]. Additionally, the myofibrillar protein oxidation also resulted in color changes of protein gels. In this regard, protein oxidation decreases the whiteness of gels, and this was related to the carbonylation process, which produces a more turbid appearance and, thus, the color changes [123].

On the other hand, it is well known that oxidative and proteolytic reactions play an important role in the release of volatile compounds, which have a direct impact on the flavor and aromas of muscle foods [9,124]. Therefore, it is expected that protein oxidation, which could affect both types of reactions, influences the final flavor of muscle foods [2]. Some oxidized protein products, such as protein carbonyls, α-aminoadipic, and γ-glutamic semialdehydes determine the formation of Strecker aldehydes from leucine and isoleucine, which are involved in the formation of the final aromatic compounds from the Maillard reaction [117]. Additionally, the oxidation of aromatic amino acid released end products and advanced glycosylation end products could also affect the muscle food aroma [121]. Thus, the simultaneous existence of proteolysis and protein oxidation (especially on maturated and ripened further processed meat and fish products) could have a determinant influence on the final aroma of these products, via the formation of Strecker aldehydes [117]. Moreover, the oxidation of sulfur-containing amino acids is also related with the production of off-flavors [101].

Another important techno-functional property of the proteins in muscle foods is their water-holding capacity. This parameter is also related to textural changes in these foods. Most water (about 85%) in muscle foods is held within the myofibrillar matrix (composed of actin and myosin) [117,125,126], and thus the changes in the myofibrils volume (e.g., filament charges and structural constraints) were proposed to explain the relationship between water-holding capacity and protein oxidation [112,113]. Oxidative modifications can lead to altered protein charges [112] due to the carbonylation process, which involves histidine, but also lysine and arginine residues (in positively charged forms) [14]. Thus, these amino acids lose their positive charges upon oxidation, which affect the protein charges, and result in an increase of net negative charges [117,127]. This would, in turn, increase the electrostatic repulsion between myofilaments and increase the swelling pressure and volume of myofibrils [113], and contribute to increasing water-holding capacity of the muscle system. In contrast, protein oxidation also increases the cross-linked structures within and between proteins. This process increases the constraints and produces a reduction in the ability of myofibrils to swell [113]. Therefore, cross-linking strengthens the protein structure, which should result in an overall decrease in water-holding capacity. Additionally, the inhibition of proteolytic reactions (as commented above) with protein oxidation could negatively affect the water holding capacity. This is supported by several research studies that have reported a positive relationship between proteolysis and the water-holding capacity of muscle foods [128,129]. Recently, in a similar way to proteolysis, it was suggested that moderate oxidation favors ordered protein interactions which enhance the functionality of protein foods, while excessive levels of oxidation promote random protein aggregation and reduce protein functionality [7,111]. So, the dose-dependent behavior of protein functionality could partially explain the effect of oxidation on the water-holding properties of proteins. Moreover, the oxidative processes alter the ability of proteins to bind with water molecules through hydrogen bonding, electrostatic repulsion, or capillary action [41]. Consequently, the influence of protein oxidation on water-holding capacity is a balance of promoting (e.g., increase of myofibrils swelling and negative net charges) and inhibiting factors (e.g., cross-linking or reduction of proteolysis) [111,112,128], while also being dependent on the degree of protein oxidation.

In addition to water-holding capacity, protein oxidation also influences other techno-functional parameters such as protein solubility, gelation, and emulsifying properties [2,3]. In this sense, high levels of protein oxidation result in a decrease in protein solubility, due to protein denaturation and precipitation [3]. In contrast, protein oxidation could improve both the gelation and emulsification properties of proteins [3,123]. The gels produced from moderately-oxidized proteins also presented a better shear force and true strength, which are important parameters when manufacturing emulsified muscle food products. In fact, the ability of muscle foods to form protein gels is one of the most important functionalities for processed meat and fish products [123]. The main explanation for the improvement of gel-forming ability with moderately-oxidized proteins is related to the increased formation of cross-link structures between proteins and polypeptides, and the stabilization of other non-covalent bonds, which decrease the mobility of the gel network and stabilize the gel matrix [3]. Additionally, moderate protein oxidation also favors the creation of a dense network with a homogeneous distribution of pores [123]. These aspects were shown by Zhou et al. [123], who concluded that the dynamic rheological properties of myofibrillar protein changed with protein oxidation, and that the physico-chemical changes (mainly disulfide-bond formation) might facilitate strengthening of gel networks during thermal gelation. In contrast, it is important to highlight that excessive levels of protein oxidation has inverse consequences, and actually impairs the gelling capacity [123,127].

In addition to the aforementioned physico-chemical changes, protein oxidation also has a significant role in the loss of nutritional quality of muscle foods [111]. First, it is important to highlight that the quality of protein (from a nutritional point of view) could be defined as the ability to achieve specific metabolic functions, which are related to the composition of amino acids, peptide sequence, native structure, and bioavailability [4]. Therefore, the most evident consequence of protein oxidation on the nutritional quality of muscle foods is the loss of essential nutrients (i.e., amino acids) [111] and a considerable change in the amino acid profile [117]. Some amino acids are particularly susceptible to oxidative degradation. In this sense, it is well known that cysteine, tyrosine, phenylalanine, tryptophan, histidine, proline, arginine, lysine, and methionine are highly vulnerable to ROS [2]. Among them, histidine, arginine, cysteine, phenylalanine, tryptophan, lysine, and methionine are considered essential (i.e., indispensable) or semi-essential amino acids, and thus they should be supplied in the human diet [3]. Consequently, the irreversible oxidative modification of essential amino acids by carbonylation produces a clear detrimental impact on the nutritional value of muscle foods [2,4,117]. Moreover, oxidative modifications of these compounds can also limit their bioavailability [2].

Additionally, as discussed at the beginning of this section, several compounds derived from protein oxidation are considered harmful (e.g., heterocyclic aromatic amines or advanced glycation end products, α-aminoadipic semialdehyde, kynurenines, etc.), and thus protein oxidation has been shown to generate potentially toxic compounds, which undoubtedly reduce the nutritional quality of muscle foods [98,111]. Moreover, the oxidized amino acids that resist digestion could be utilized by gut microbiota and turned into mutagenic compounds such as biogenic amines, ammonia, cresol, and indole [6,111], each of which has been associated with increased risk of colon cancer [117].

Although protein digestibility is a complex mechanism, oxidation could affect it. There is controversy about the effect of protein oxidation on protein susceptibility to digestive enzymes [4]. In this sense, some authors stated that oxidative reactions impair protein digestibility [3,98]. However, it is important to note that the influence of protein oxidation on digestibility is dependent on the level (or severity) of oxidation. As reported before, moderate oxidation could promote the unfolding of protein, and thus increase the accessibility of proteases of the digestive tract (e.g., pepsin, trypsin, and alpha-chymotrypsin) to these proteins and favor their digestibility [4,130]. However, excessive oxidation produces severe polymerization and aggregation, which impairs protein digestibility [2,130,131,132]. The oxidative degradation of specific amino acid side chains would chemically and physically alter the recognition sites and reduce the digestibility of these proteins [4,117], since oxidized proteins are resistant from being properly hydrolyzed by pancreatic and digestive enzymes [4,88]. This is despite, as a general statement, the results showed a direct relationship between the loss of protein digestibility and oxidative protein damages [117,130]. In fact, a recent study found a high correlation between protein carbonylation and digestibility values of muscle foods, which confirm both the loss of essential amino acids and the altered digestibility of oxidized proteins [88].

## 6. Conclusions

In conclusion, protein oxidation induces several molecular changes in proteins, including chemical modifications and structural changes, which result in significant alterations to the quality of muscle foods. In this sense, physico-chemical and techno-functional properties (e.g., texture, color, flavor, water-holding capacity, protein solubility, protein gelling ability, and protein emulsification ability), as well as the nutritional quality (e.g., loss of essential amino acids, production of toxic compounds, and decrease in bioavailability and digestibility), could be highly affected by the multiple oxidative reactions of proteins. It is important to highlight that some mechanisms, including the interactions of lipid and protein oxidized products, and the molecular consequences of protein oxidation, are not fully elucidated by the current body of research. The development of new analytical techniques and specific studies on protein oxidation will undoubtedly contribute to a better understanding for many of these reactions. Therefore, the relationship between protein oxidation and the quality of muscle foods requires more investigation. Finally, understanding the mechanisms of protein oxidation and their consequences on the quality of muscle foods and their properties will enable the food industry to manufacture higher quality and safer products in the future.

## Figures and Tables

**Table 2:** Studies evaluating protein oxidation in different muscle foods.

Indicator	Food Product	Method	Results	Ref.
Carbonyls content ^A^	Chicken breast	DNPH	0.2−2.9	[87]
	Ready-to-eat chicken patties		5−19	[88]
	Fermented sausages		1.5−4.5	[89]
	Raw meat		2.5	[90]
	Dry-cured ham		8	
	Dry-cured loin		8	
	Dry-cured sausage		9	
	Cooked sausage		13	
	Pork sausages		2.36−3.35	[91]
	Sturgeon fillets		2.1−10.6	[88]
	Rainbow trout		2.8−2.9	[92]
	Horse mackerel fillets		1.7−7.2	[93]
	Pacific white shrimp		3−9.5	[94]
Quantification of specific carbonyls ^A^	Fermented sausages	HPLC-FLD	AAS: 0.17−0.35; GGS: 0.09−0.11	[89]
	Porcine patties		AAS: 0.27−1.09; AAA: 0.56−0.84	[95]
	Dry-cured loins		0.13−1.10	[58]
	Beef patties		AAS: 131.07−857.61; AAA: 5.35−21.3	[66]
	Pork sausages		AAS: 1.47−1.57; GGS: 0.23−0.28	[91]
	Raw meat	LC–ESI–MS	AAS: 27; GGS: 30	[90]
	Dry-cured ham		AAS: 23; GGS: 150	
	Dry-cured loin		AAS: 23; GGS: 115	
	Dry-cured sausage		AAS: 22; GGS: 120	
	Cooked sausage		AAS: 28; GGS: 60	
Free thiol content ^B^	Rabbit meat	DTNB	24.3−34.7	[96]
	Sturgeon fillets		17−35	[97]
	Sturgeon fillets		0.3−0.8	[98]
	Horse mackerel fillets		98.6−124.4	[93]
	Silver carp		5.3−7.2	[99]
	Pacific white shrimp		28−42	[94]
	Chicken breast	4-DPS	15−16	[87]
	Jerky chicken		25−55	[100]
	Ready-to-eat chicken patties		21−52	[88]
	Pork sausages		14.80−21.80	[91]
	Ground beef		26.5−37.6	[99]
Tryptophan content ^C^	Chicken breast	FS	11−13	[87]
	Jerky chicken		8−10 *	[100]
	Porcine patties		0.14−0.77	[95]
	Beef patties		0.16−2.54	[66]
	Ready-to-eat chicken patties		100−170	[88]
Cross linking proteins	Rabbit meat	SDS-PAGE	Reduced myofibrillar protein content	[96]
	Ground beef		Oxidized proteins	[99]
	Sturgeon fillets		Reduced myofibrillar protein content	[97]
	Sturgeon fillets		Reduced myofibrillar protein content	[98]
	Pacific white shrimp		Reduced myofibrillar protein content	[94]
	Rainbow trout	SDS-PAGE and Immunoblotting	Oxidized proteins	[92]
	Horse mackerel fillets		Oxidized proteins	[93]
Cross linking proteins- Disulphide bonds ^D^	Jerky chicken	Total-Free thiol difference	12−27	[100]
	Ready-to-eat chicken patties		5−17	[88]
	Ground beef		7.0−11.5	[99]
Cross linking proteins- Schiff bases ^E^	Chicken breast	FS	6−7	[87]
	Dry-cured loins		420	[58]
	Jerky chicken		600−780	[100]
	Porcine patties		23.7−169.0	[95]
	Beef patties		3383−992	[66]
	Ready-to-eat chicken patties		410−900	[88]

^A^ Data expressed as nmol carbonyls/mg protein. ^B^ Data expressed as µmol thiols/mg sample. ^C^ Data expressed as N-acetil-L-tryptophan amide equivalents per 100 g of sample. * N-acetil-L-tryptophan amide equivalents per g of protein. ^D^ Data expressed as nmol/mg protein. ^E^ Data expressed as fluorescence intensity. Abbreviations: AAS, α-aminoadipic semialdehydes; GGS, γ-glutamic semialdehydes; AAA, aminoadipic acid; DNPH, 2,4- Dinitrophenylhydrazine; DTNB, 5′5-dithiobis (2-nitrobenzoate); 4-DPS, 4,4′-dithiodipyridine; HPLC-FLD, high performance liquid chromatography-fluorescence detection; LC-ESI-MS, Liquid Chromatography Electrospray Ionization Tandem Mass Spectrometric; FS, Fluorescence spectroscopy; SDS-PAGE, sodium dodecyl sulphate–polyacrylamide gel electrophoresis.

## Data Availability

All data are available in the manuscript.
